# Mitochondrial DNA paradox: sex-specific genetic structure in a marine mussel – despite maternal inheritance and passive dispersal

**DOI:** 10.1186/1471-2156-13-45

**Published:** 2012-06-13

**Authors:** Peter R Teske, Isabelle Papadopoulos, Nigel P Barker, Christopher D McQuaid

**Affiliations:** 1 Department of Zoology and Entomology, Rhodes University, Grahamstown, 6140, South Africa; 2 Molecular Ecology and Systematics Group, Botany Department, Rhodes University, Grahamstown, 6140, South Africa; 3 Molecular Ecology Laboratory, School of Biological Sciences, Flinders University, Adelaide, South Australia, 5001, Australia

## Abstract

**Background:**

When genetic structure is identified using mitochondrial DNA (mtDNA), but no structure is identified using biparentally-inherited nuclear DNA, the discordance is often attributed to differences in dispersal potential between the sexes.

**Results:**

We sampled the intertidal rocky shore mussel *Perna perna* in a South African bay and along the nearby open coast, and sequenced maternally-inherited mtDNA (there is no evidence for paternally-inherited mtDNA in this species) and a biparentally-inherited marker. By treating males and females as different populations, we identified significant genetic structure on the basis of mtDNA data in the females only.

**Conclusions:**

This is the first study to report sex-specific differences in genetic structure based on matrilineally-inherited mtDNA in a passively dispersing species that lacks social structure or sexual dimorphism. The observed pattern most likely stems from females being more vulnerable to selection in habitats from which they did not originate, which also manifests itself in a male-biased sex ratio. Our results have three important implications for the interpretation of population genetic data. First, even when mtDNA is inherited exclusively in the female line, it also contains information about males. For that reason, using it to identify sex-specific differences in genetic structure by contrasting it with biparentally-inherited markers is problematic. Second, the fact that sex-specific differences were found in a passively dispersing species in which sex-biased dispersal is unlikely highlights the fact that significant genetic structure is not necessarily a function of low dispersal potential or physical barriers. Third, even though mtDNA is typically used to study historical demographic processes, it also contains information about contemporary processes. Higher survival rates of males in non-native habitats can erase the genetic structure present in their mothers within a single generation.

## Background

Genetic markers with sex-specific inheritance have long been used to study differences in genetic structure between males and females. Numerous studies have used maternally-inherited mitochondrial DNA (mtDNA) in conjunction with biparentally-inherited nuclear DNA markers [1-7], and discrepancies between results from the two types of markers have been interpreted as the result of sex-specific differences in dispersal.

For marine invertebrates, sex-biased dispersal is not usually considered because dispersal in most of these species takes place either by means of planktonic larvae [8] or by association of adults or egg masses with floating objects [9]. Nonetheless, sex-specific differences in genetic structure were found in the marine mussel *Mytilus edulis*[10], despite the fact that it has microscopic larvae with extremely limited swimming abilities and presumably passive dispersal. The family Mytilidae includes 33 genera [11] and in five of these, including *Mytilus*[12,13], doubly uniparental inheritance (DUI [14]) has been identified. Males have both a male (M-mtDNA) and a female (F-mtDNA) mitochondrial genome, whereas females only have a female genome. Levels of trans-Atlantic gene flow differ for the two genomes of *M. edulis*, with genetic interchange being evident for F-mtDNA but not for M-mtDNA [10]. A genetic barrier for M-mtDNA exchange (possibly linked to DUI) was considered to be a more likely explanation for the observed pattern than gender-specific differences in larval dispersal capability.

A recent study comparing genetic structure in the brown mussel (*Perna perna*) between several South African coastal sites and bays [15] identified a surprisingly large amount of mtDNA-based genetic structure at this relatively small geographic scale (10-100s km). While there was no structure between coastal regions, sites within bays were not only genetically distinct from those on the open coast, but also from each other. This genetic pattern was interpreted as being the result of highly asymmetrical levels of larval dispersal between coastal habitats and bays. The study included only samples from females to avoid complications associated with DUI, as mtDNA genome-specific primers such as those used for *Mytilus*[10] are not available for the genus *Perna*. However, as levels of gene flow in marine mussels may differ between male and female mitochondrial genomes [10], it is ill advised to ignore the males. In the present study, we explored the previously identified genetic structure in South African *P. perna*[15] in more detail by sequencing both a mitochondrial (COI) and a nuclear (ITS-2) genetic marker from female and male individuals.

## Results

A total of 374 DNA sequences were generated, including 158 COI sequences and 216 ITS-2 sequences. These were submitted to GenBank (accession numbers JX075516 - JX075889). Complete data-sets of aligned sequences are available in the following additional files: COI: Additional File 1; ITS-2: Additional File 2.

While many individuals had two ITS-2 alleles (some nucleotide positions had two electropherogram peaks, which indicates that a single version of the marker amplified), there was no evidence for more than one COI allele in either male or female mussels. Sequences from male and female mitochondrial genomes tend to be phylogenetically distinct [10], but we identified only a single evolutionary lineage (not shown). Moreover, we did not find differentiation between the COI sequences of males and females when treating these as distinct populations (Φ_ST_ = −0.003; *p* = 0.550; 95% confidence interval: -0.005 – 0.000). These results indicate that no M-mtDNA amplified in the males.

Mitochondrial COI sequence data had approximately twice as much haplotype diversity as ITS-2 data (Table 1) and the marker was overall more informative (mean uncorrected p-distances: COI = 0.009, ITS-2 = 0.003; maximum p-distances: COI = 0.043, ITS-2 = 0.023). For both sexes, the number of COI haplotypes recovered from Algoa Bay, South Africa, was slightly higher than the number recovered from the adjacent open coast. There was a sex-specific difference for ITS-2, with the data-sets for males from both habitats having a larger number of rare alleles than those for females.

**Table 1:** **Genetic diversity of brown mussels,*Perna perna***

**Genetic Marker**	**Group**	***N***	***H* (corrected)**	***h* (± S.D.)**
COI	Females Coast	40	15	0.753 ± 0.066
	Females Bay	40	19	0.924 ± 0.034
	Males Coast	38	15 (16)	0.844 ± 0.050
	Males Bay	40	21	0.879 ± 0.056
ITS-2	Females Coast	22	5 (7)	0.318 ± 0.083
	Females Bay	30	4 (4)	0.381 ± 0.057
	Males Coast	24	8 (11)	0.536 ± 0.077
	Males Bay	32	9	0.496 ± 0.056

*N* = number of individuals sequenced (this number corresponds to the number of COI sequences and half of the ITS-2 sequences generated), *H* = number of haplotypes recovered (including values corrected for differences in sample size using *N*_max_ = 40 for COI and *N*_max_ = 32 for ITS-2), *h* = haplotype or gene diversity.

**Figure 1:**
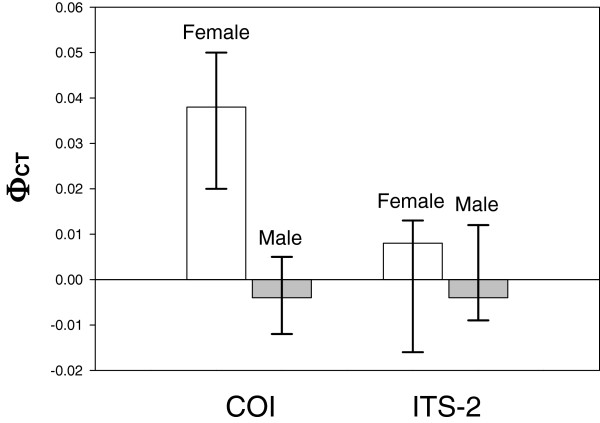
**Genetic structure in female and male brown mussels,*Perna perna.*** Magnitude of the statistic Φ_CT_ among coastal and bay populations of female and male brown mussels based on mitochondrial COI and nuclear ITS-2 data. Whiskers are 95% confidence intervals.

Female *P. perna* showed marginally significant genetic structure between the bay and the open coast on the basis of mtDNA COI sequence data (*p* = 0.049), but there was no structure between males from the two areas (*p* = 0.537) (Figure 1). Based on confidence intervals, the Φ_CT_ value for males was both significantly lower than that for females and not signficantly different from zero. No genetic structure was found for either females (*p* = 0.279) or males (*p* = 0.444) on the basis of the ITS-2 data. Although Φ_CT_ was also greater for females, the difference was not significant, and neither estimate was significantly different from zero.

## Discussion

The larger data-set of COI sequences generated in this study confirms the previous results for female *Perna perna*[15], but interestingly, no genetic structure was found in the males. These sex-specific differences are unlikely to be an artifact of two mitochondrial genomes being subject to differential evolutionary constraints *sensu* 10], because this should have resulted in two copies of COI amplifying with the universal primers used here. Maternally- and paternally-inherited mtDNA genomes are usually highly distinct [10,16,17], but the fact that we did not even find genetic structure between males and females (which would be expected if there was a recent masculinisation of female-transmitted mtDNA) suggests that *P. perna* does not exhibit DUI (see also [18]) and so can serve as a model for other marine invertebrates in which the sexes are separate and mtDNA is inherited only in the female line.

Although more ITS-2 alleles were recovered for males than for females, no genetic structure was found with this genetic marker for either sex. Possible reasons for this include less genetic variation and the larger effective population size of the nuclear genome [19].

Our results have important implications for interpreting genetic structure and highlight the value of analysing genetic data from males and females separately. Surprisingly, in many studies on species with breeding behaviours that differ between the sexes, genetic data from males and females have been combined [2-4]. Gender-specific differences in genetic structure based on maternally-inherited mtDNA sequences have so far only been reported for social mammals and have been attributed to the sexes exhibiting different dispersal patterns [20-22]. As males cannot pass their mitochondrial genome to the next generation, their lack of genetic structure implies considerably greater contemporary gene flow than in the females.

Male-biased gene flow in *P. perna* (which lacks both social structure and external sexual dimorphism) could be explained by sex-specific differences in larval behaviour, including differences in the way this behaviour influences their position in the water column [23], and differences in larval development time. Although larval behaviour in *P. perna* has not been investigated, this seems unlikely as larvae of this species disperse as passive particles [24], and we are aware of no studies reporting sex-specific differences in the larval behaviour of mussels. Alternatively, as the magnitude of genetic structure depends on effective population sizes [25], it is possible that the differences between genders in mtDNA structure are due to lower female population sizes. Preliminary data indicate that the sex ratio in *P. perna* is male-biased (coast: 2.8 males : 1 female; *n* = 103; bay: 1.2 males : 1 female, *n* = 326), so the stronger male bias on the open coast, where wave action is stronger, could reflect the negative consequences of weakened attachment strength due to greater reproductive effort by the female mussels [26]. We nonetheless consider it unlikely that the resulting reduction in female effective population size is sufficient to explain the observed genetic structure. The mtDNA diversity of males reflects that of females from the previous generation. Lack of structure suggests not only that there is a large amount of gene flow between bays and coastal habitats, but also that the pool of female larvae from the present generation is unlikely to have lower mtDNA diversity than that of the males. Instead, it is possible that in every generation, large numbers of females are eliminated because they are less likely to survive in habitats from which they did not originate, thus reinforcing genetic differentiation between habitats.

Females of bay populations tend to have a larger number of private haplotypes than those of coastal populations [15]. Hence, even though there are no distinct habitat-linked mtDNA lineages of *P. perna*, bay individuals having certain haplotypes may be particularly vulnerable to strong wave action on the open coast. We hypothesise that females having these haplotypes expend more energy on reproduction rather than attachment, which results in an overall greater gamete output in mussels that reside in bays [27]. Sex-specific genetic structure in the mtDNA of the mussel *P. perna* may therefore stem not from differential dispersal of the sexes, but from sex- and habitat-specific differences in reproductive effort and the effects of differential selection pressures between bays and the open coast.

## Conclusions

The fact that female-only mtDNA structure can be present in a species in which sex-biased dispersal is highly unlikely indicates that female philopatry and male roving [20-22] do not necessarily need to be invoked to explain such patterns. The lack of genetic structure in male mussels further challenges the notion that significant genetic structure in marine organisms must be the result of dispersal barriers, such as upwelling cells or coastal heterogeneity [15,28].

Mitochondrial DNA is by far the most frequently used locus in phylogeographic studies [29], but its role as a marker that can be used to detect sex-specific differences in genetic structure remains to be fully appreciated. When studying genetic structure in closely related populations, mitochondrial DNA is undoubtedly an inferior tool compared to microsatellites, which are both highly variable and biparentally inherited. However, designing microsatellite libraries has been problematic in several invertebrate species [30,31], and mtDNA could therefore serve as a simple alternative to these markers that may be applicable to a wide range of taxa. Furthermore, while the high mutation rate of microsatellites makes these the markers of choice for studying near-contemporary demographic events, the finding that mtDNA structure that must have been present in the mothers of the male individuals studied here was lost only a generation later is an interesting example of how mtDNA can contain present-day demographic information.

## Methods

### Sampling sites

*Perna perna* was sampled at four sites in Algoa Bay (GPS coordinates: 33.89611°S, 25.62133°E; 33.89696°S, 25.62101°E; 33.89524°S, 25.62159°E; 33.88181°S, 25.62709°E) and four sites along the adjacent open coast (34.03662°S, 25.64517°E; 34.04371°S, 25.54786°E; 34.04343°S, 25.54703°E; 34.04400°S, 25.55092°E).

### Identification of gender

Individuals with white gonads were considered to be male and those with orange gonads to be female. We excluded smaller individuals lacking gonads and those in which the difference was not obvious, which can be a consequence of infection by bucephalid trematodes [32]. It has recently been shown that in marine mussels of the genus *Mytilus*, the gonads of males can become orange in response to environmental stress [33]. Although this has not yet been reported in *P. perna*, we considered it necessary to confirm that gonad colour can be used to distinguish between males and females of this species. We selected 20 individuals with white gonads and 20 individuals with orange gonads, dehydrated the tissues and embedded them in paraffin wax, and sectioned them to a thickness of approximately 5 µm. Histological sections were then stained with haematoxylin-eosin and examined under a compound microscope, and the sex was determined by the presence of ova or spermatids. We found that all individuals with white gonads were male and all individuals with orange gonads female, and thus conclude that gonad colour is a reliable indicator of gender in our samples of *P. perna*.

### Amplification of genetic markers

DNA was extracted from gonad tissue using a modified CTAB protocol [34]. The COI gene was amplified as described previously [15]. Samples that did not amplify on the second attempt were excluded, resulting in a final data-set comprising 158 sequences (Table 1). In addition, a portion of ITS-2 was amplified using primers ITS5 (5’-GGA AGT AAA AGT CGT AAC AAG G-3’) and ITS28 (5’-CGC CGT TAC TAG GGG AAT CCT TGT AA-3’) [35]. Although amplification was reliable, there were several complications with this marker. First, in some cases two bands amplified, both of which were excised from the agarose gel and the PCR products sequenced separately. BLAST searches [36] revealed that the additional bands were ITS-2 of trematode parasites. Many of the ITS-2 sequences of *P. perna* were not usable because they contained long sections with multiple electropherogram peaks that were difficult to interpret and may have resulted from a combination of secondary structure problems, slip-strand mispairing when sequencing through a long AT-array, and length differences between alleles in the case of individuals that were heterozygous at this locus. For that reason, we decided to use only a relatively short segment (216 bp) that started on the 3’ side of the AT-array and that could be unequivocally scored in a total of 108 individuals. In these sequences, a particular nucleotide position was considered to be heterozygous when the intensity of a secondary peak in the chromatograms was at least 25% of that of the primary peak. While this threshold is slightly above that of 20% typically used in the literature [e.g. [37,38], the fact that no smaller secondary peaks were found suggests that this did not result in any loss of genetic information. The individual alleles comprising heterozygous ITS-2 sequences were deduced using the PHASE v2.1 algorithm [39,40] implemented in DnaSP v5.10.01 [41]. We specified 10 000 iterations, a thinning interval of 10 and a burn-in of 1000 iterations, with default options specified for all other parameters. All sequences could be fully resolved.

### Genetic analyses

Genetic distances among sequences were calculated in MEGA v5.05 [42]. Haplotype diversity of each combination of population and gender was estimated in ARLEQUIN v3.5.1.2 [43] and genetic structure was investigated by conducting an Analysis of Molecular Variance (AMOVA) [44] in GENODIVE v2.0b20 [45] using a matrix of uncorrected pairwise differences between haplotypes. Traditional fixation indices such as *F*_ST_[46] and Φ_ST_[47] have been criticised for being strongly affected by within-population diversity, and alternatives such as *G*_ST_[48] and *D*[49] have been developed. However, DNA sequence data contains information on the evolutionary relationships between haplotypes, and the alternative statistics cannot incorporate such information [50]. We based our results on the fixation index Φ_CT_, which in this case represents genetic structure between a group including all coastal sites vs. a second group that includes all sites within the bay, and on Φ_ST_, which represents genetic structure between all male and all female individuals (COI sequences only). Ninety-five percent confidence intervals were obtained by generating 1000 bootstrap replications over variable positions.

## Competing interests

The author(s) declare that they have no competing interests.

## Authors’ contributions

CDM and NPB conceived the study. PRT and IP collected samples and generated DNA sequence data. PRT analysed the genetic data. CDM investigated sex-ratios and gonad-based indentification of gender. PRT, IP and CDM wrote the paper. All authors read and approved the final version.

## Supplementary Material

Additional file 1 COI sequences. Click here for file

Additional file 2 ITS-2 sequences. Click here for file
